# Balloon Pulmonary Angioplasty with Stent Implantation as a Treatment of Proximal Chronic Thromboembolic Pulmonary Hypertension

**DOI:** 10.3390/diagnostics10060363

**Published:** 2020-06-01

**Authors:** Szymon Darocha, Radosław Pietura, Marta Banaszkiewicz, Arkadiusz Pietrasik, Łukasz Kownacki, Adam Torbicki, Marcin Kurzyna

**Affiliations:** 1 Department of Pulmonary Circulation, Thromboembolic Diseases and Cardiology, Centre of Postgraduate Medical Education, Fryderyk Chopin Hospital in European Health Center Otwock, 05-400 Otwock, Poland; marta.banaszkiewicz@gmail.com (M.B.); adam.torbicki@ecz-otwock.pl (A.T.); marcin.kurzyna@ecz-otwock.pl (M.K.); 2 Department of Radiography, Medical University of Lublin, 20-081 Lublin, Poland; radoslawpietura@poczta.onet.pl; 3 1st Chair and Department of Cardiology, Medical University of Warsaw, 02-087 Warsaw, Poland; apietrasik@tlen.pl; 4 Department of Radiology, European Health Center Otwock, 05-400 Otwock, Poland; luke@mrlab.pl

**Keywords:** chronic thromboembolic pulmonary hypertension, balloon pulmonary angioplasty, stent implantation

## Abstract

We present a case of a 67-year-old female with proximal chronic thromboembolic pulmonary hypertension (CTEPH), disqualified from pulmonary endarterectomy due to multiple comorbidities and high risk-to-benefit ratio as assessed by multidisciplinary CTEPH team. She was referred for balloon pulmonary angioplasty (BPA) and underwent three sessions with balloon catheters up to 8 mm diameter. During the second procedure, the elastic recoil phenomenon was observed in the treated post-thrombotic lesion of the right lower lobe artery, which made the balloon angioplasty ineffective. It was decided to implant a self-expanding stent for the prevention of restenosis. The procedure resulted in significant improvement of regional perfusion, as confirmed by control angiography. We feel that it contributed to the significant improvement of hemodynamic parameters and exercise capacity, as assessed three months after the last BPA procedure. In conclusion, pulmonary artery stenting may be an option in proximal CTEPH when elastic recoil phenomenon makes balloon angioplasty of a large vessel ineffective.

## 1. Introduction

Pulmonary endarterectomy (PEA) is the “gold standard” treatment for chronic thromboembolic pulmonary hypertension (CTEPH) [1,2]. Balloon pulmonary angioplasty (BPA) is routinely performed in patients technically inoperable because of the distal type of CTEPH [3]. However, increased experience has allowed BPA to also be performed in proximal disease assessed as technically operable but rejected from PEA because of comorbidities or patient’s refusal, as well as in patients with pulmonary hypertension persisting after ineffective PEA [4,5,6].

## 2. Case Report

A 67-year-old female was admitted for management of suspected CTEPH after an episode of acute pulmonary embolism two years earlier. She had a history of surgical mitral valve replacement, tricuspid valve annuloplasty, and pacemaker implantation. Additionally, she had diabetes, persistent atrial fibrillation, arterial hypertension, and Parkinson’s disease. She was on chronic antithrombotic therapy with vitamin K antagonist and home oxygen supplementation. This research study obtained the patient’s consent.

On admission, she was in the IV World Health Organization (WHO) functional class and was not able to perform the six-minute walk test (6MWT). N-terminal pro-B-type natriuretic peptide (NT-proBNP) serum concentration was increased to 4216 pg/mL. Right heart catheterization confirmed precapillary pulmonary hypertension, with pulmonary artery pressure (mPAP) of 32 mm Hg, cardiac index (CI) of 2.31 L/min*m^2^, and pulmonary vascular resistance (PVR) of 4.74 Wood’s units (Table 1).

**Figure 1:**
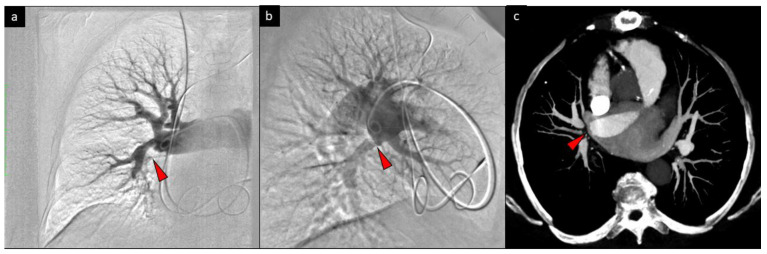
Angiography of the right pulmonary artery with a proximal thromboembolic lesion (marked by the red arrow) in the anteroposterior view (**a**), lateral view (**b**), and computed tomography (**c**).

Computed tomography pulmonary angiography and pulmonary angiography confirmed proximal thromboembolic lesions (Figure 1).

**Figure 2:**
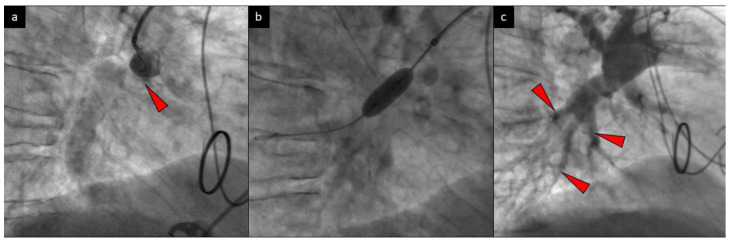
Selective angiography of the right pulmonary artery presents a subtotal occlusion (arrow) of segments A9 and A10 (**a**) and BPA of the lesion with a 7.0 mm balloon catheter (**b**). Control angiography with the improvement of distal perfusion (arrows) is shown (**c**).

**Figure 3:**
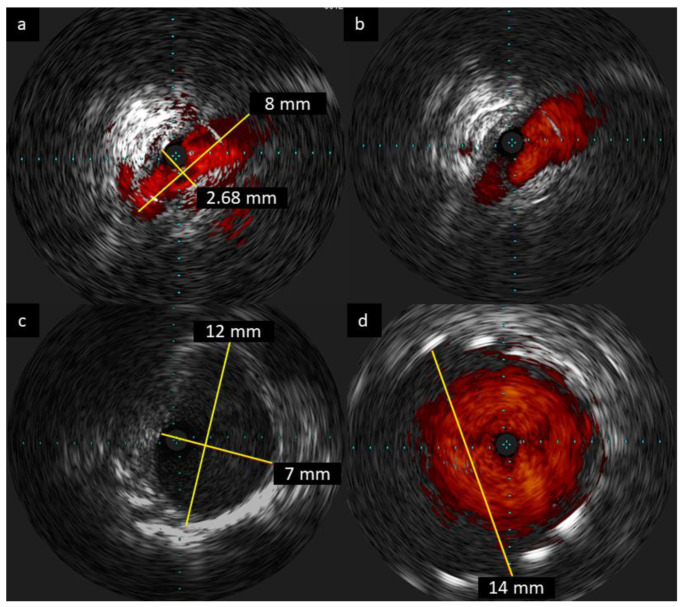
The intravascular ultrasound (IVUS) examination of right segmental A9/A10 pulmonary artery. Critical stenosis was confirmed in the first BPA session (**a**). The second BPA session revealed elastic recoil phenomenon after the use of increasing diameters (4.0 mm, 7.0 mm, and 8.0 mm) of balloon catheters (**b**). Distal A9/A10 reference diameter (**c**) for proper stent selection in third BPA session. Final result after 14 × 30 mm stent implantation (**d**).

The patient’s case was analyzed by the multidisciplinary CTEPH team and was not qualified for PEA due to the high risk-to-benefit ratio caused by numerous comorbidities. Because of her debilitating disease, BPA was offered to the patient, and the first session was performed within the right proximal pulmonary artery. Lesions within A9/A10 segmental pulmonary artery were dilated with 4.0 mm × 20 mm (Pantera Pro, Biotronik, Bülach, Switzerland) and 7.0 mm × 20 mm (Sterling^TM^, Boston Scientific, Quincy, MA, USA) balloon catheters under the guidance of the intravascular ultrasound (IVUS) (Figure 2, Figure 3a).

During the second BPA session, left segmental A4, A5, and A6 pulmonary arteries were successfully dilated with 3.0 mm × 15 mm (Pantera Pro, Biotronik, Bülach, Switzerland) balloon catheters and proximal segmental A9 of left pulmonary artery was dilated with a balloon 8.0 mm catheter (Sterling^TM^, Boston Scientific, Quincy, MA, USA). Selective angiography of the previously treated trunk of the right A9/A10 segmental pulmonary artery revealed the presence of its restenosis. The BPA of the lesion was attempted again with a 8.0 mm × 20 mm balloon catheter (Sterling^TM^, Boston Scientific, Quincy, MA, USA) without any significant angiographic improvement. Due to this unsatisfactory result, the team decided to perform stent implantation during the next BPA procedure.

During the third BPA session, based on selective angiography and IVUS guidance (Figure 3b–d), a self-expanding 14 mm × 30 mm stent (EPIC^TM^, Boston Scientific, Quincy, MA, USA) was deployed in the proximal trunk of A9/A10 segmental arteries and post-dilated with a 12 mm × 40 mm balloon catheter (POWERFLEX, Cordis, Miami Lakes, FL, USA) at 12 atmospheres. 

**Figure 4:**
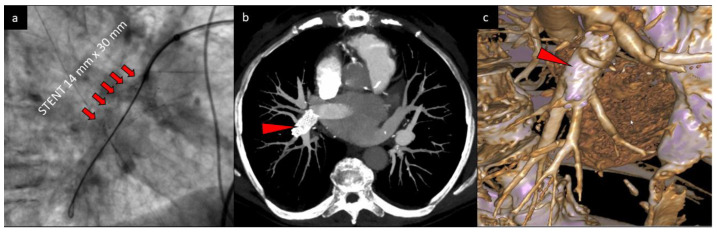
The final result of BPA and stent implantation (marked by red arrows) presented in fluoroscopy (**a**), computed tomography (**b**), and 3D reconstruction (**c**).

The follow-up examination revealed normally expanded stent and maintained patency of distal branches (Figure 4). The intervention was accomplished without any complications.

Follow-up examination performed three months after the final BPA session presented a significant reduction of mPAP to 23 mm Hg and normalization of PVR at 2.25 Wood’s units. The patient improved to III WHO functional class, and NT-proBNP decreased to 2109 pg/mL. The patient performed 6MWT with a distance of 248 m (Table 1).

## 3. Discussion

To the best of our knowledge, this is the first report of stent implantation as a part of a planned strategy of management of a CTEPH patient. According to the character of the thromboembolic lesions, there is no need for routine stent implantation in CTEPH patients undergoing BPA [7,8]. However, the thromboembolic lesions in some patients are challenging to dilate permanently because they may be bulky and fibrotic. Especially those located proximally and forming a compact tissue may exhibit elastic recoil when treated with balloon angioplasty. For this reason, slightly oversized balloons are used to break the structure of the thrombus, which causes an increased risk of vessel injury and rupture. Rescue implantation of a covered stent effectively protects the ruptured vessel [9,10,11], and this is currently the only acknowledged indication for stent implantation in CTEPH [12,13]. 

We decided to present this novel indication for stenting the pulmonary artery, with the aim of protecting against restenosis of proximal lesions. We described a case of highly symptomatic, technically operable CTEPH at a high risk of PEA, which was referred to BPA. The strategy of adding to two regular BPA sessions elective stent implantation to the most narrowed lower lobe artery, which presented elastic recoil after previous BPA, resulted in functional, hemodynamic, and serum biomarker improvement (Table 1). The patient remains in stable condition one year after the last BPA-with-stenting procedure.

We decided to use a stepwise technique of balloon catheters of increasing diameter (4.0 mm, 7.0 mm, and 8.0 mm) with the intention of reducing the risk of reperfusion pulmonary injury. We believe that the strategy of increasing the diameter of balloon catheter via several separate procedures may reduce the risk of this life threatening complication, as was reported previously by our team [14]. When we noticed that implemented strategy did not result in any improvement of the morphology of the thromboembolic lesion due to the elastic recoil phenomenon, the decision was not to increase the size of balloon catheter but to use a stent. In this procedure, we based our measurements on a distal reference of 12 mm measured at angiography and computed tomography and finally confirmed in IVUS (Figure 3c,d). We decided to use a slightly oversized self-expanding stent in order to obtain appropriate adherence across the entire length of the stent. Finally, the strategy resulted in optimal stent implantation without significant malapposition (see Video S1).

In conclusion, pulmonary artery stent implantation may be an option not only as a rescue procedure in case of procedural complications but also as an elective extension of BPA treatment when balloon angioplasty is incomplete or ineffective. More extensive studies are needed to define better safety, efficacy, indications, and optimal procedural standards in such CTEPH cases. Treatment options for CTEPH can be found in Table 2.

## Figures and Tables

**Table 1:** Hemodynamic and functional parameters at baseline, during consecutive BPA sessions, and at follow up.

Parameters	Baseline (12 Jan. 2018)	BPA_1_ (10 Oct. 2018)	BPA_2_ (13 Dec. 2018)	BPA_3_ + STENT (15 Jan. 2019)	Follow Up (29 Apr. 2019)
**HR** (bpm)	102	60	72	61	66
**RAP** (mmHg)	5	6	8	8	7
**mPAP** (mmHg)	32	31	23	25	23
**CI** (L/min/m^2^)	2.31	2.35	2.61	2.63	2.93
**CO** (L/min)	4.01	4.12	4.34	4.38	4.87
**PCWP** (mmHg)	13	12	12	14	12
**PVR** (Wood’s units)	4.75	4.75	2.53	2.50	2.25
**MVsat.O2** (%)	66	68	70	69	75
**AOsat.O2** (%)	95	96	98	92	95
**NT-proBNP** (pg/mL)	4216	4925	4069	3928	2109
**6MWT** (m)	-	-	-	-	248
**WHO FC**	IV	IV	III	III	III

6MWD: 6-min walk distance; AOsat.O2: arterial oxygen saturation; NT-proBNP: N-terminal pro-B type natriuretic peptide; BPA: balloon pulmonary angioplasty; CI: cardiac index; HR: heart rate; mPAP: mean pulmonary artery pressure; MVsatO2: mixed venous oxygen saturation; PAWP: pulmonary arterial wedge pressure; PVR: pulmonary vascular resistance; RAP: right atrial pressure; SVI: stroke volume index; WHO FC: World Health Organization functional class.

**Table 2:** Treatment options for chronic thromboembolic pulmonary hypertension (CTEPH).

Treatment Option	Advantages	Disadvantages
Anticoagulation	Protection against the recurrence of thromboembolic events	Increased risk of bleeding, inability to resolve fibrotic clots
PAH-specific medication	Improvement of functional capacity and hemodynamics	Side effects of some drugs, systemic hypotension
Diuretics	Reduction of symptoms of right heart failure	Hypokalemia, no impact on PVR reduction
Pulmonary endarterectomy	Reduction of symptoms and improvement of hemodynamics in proximal disease	Increased risk of complications in patients with multiple comorbidities and advanced age
Balloon pulmonary angioplasty	Reduction of symptoms and improvement of hemodynamics in distal disease	Increased risk of pulmonary injury
Stent implantation	Protection against elastic recoil phenomenon in selected cases of proximal disease	A risk of stent dislocation and vessel perforation, uncertain risk of stent thrombosis

## Supplementary Materials

The following are available online at https://www.mdpi.com/2075-4418/10/6/363/s1, Video S1: Final result of 14 × 30 mm stent implantation presented in the IVUS examination.

## Abbreviations

CTEPH	chronic thromboembolic pulmonary angioplasty
BPA	balloon pulmonary angioplasty
PEA	pulmonary endarterectomy
6MWT	six-minute walk test
NT-proBNP	N-terminal pro-B-type natriuretic peptide
mPAP	mean pulmonary artery pressure
CI	cardiac index
PVR	pulmonary vascular resistance
PAH	pulmonary arterial hypertension
